# PEGylated crushed gold shell-radiolabeled core nanoballs for in vivo tumor imaging with dual positron emission tomography and Cerenkov luminescent imaging

**DOI:** 10.1186/s12951-018-0366-x

**Published:** 2018-04-18

**Authors:** Sang Bong Lee, Dinesh Kumar, Yinghua Li, In-Kyu Lee, Sung Jin Cho, Sang Kyoon Kim, Sang-Woo Lee, Shin Young Jeong, Jaetae Lee, Yong Hyun Jeon

**Affiliations:** 1 New Drug Development Center, Daegu-Gyeongbuk Medical Innovation Foundation, Daegu, South Korea; 2 Department of Bionanosystem Engineering, Graduate School, Chonbuk National University, Jeonju, Republic of Korea; 3 Department of Pathology, Chemon Co. Ltd, 240, Nampyeong-Ro, Yangji-Myeon, Cheoin-Gu, Yongin-Si, Gyeonggi-Do 17162 Republic of Korea; 4 Department of Internal Medicine, Kyungpook National University School of Medicine, Deagu, 700-721 South Korea; 5 Laboratory Animal Center, Daegu-Gyeongbuk Medical Innovation Foundation, Daegu, 360-4 South Korea; 6 Department of Nuclear Medicine, Kyungpook National University Hospital, Daegu, 702-210 South Korea; 7 Leading-Edge Research Center for Drug Discovery and Development for Diabetes and Metabolic Disease, Kyungpook National University Hospital, Daegu, 702-210 South Korea

**Keywords:** Gold nanoparticles, Crushed gold shells, Radioactive iodine-124, Nuclear medicine imaging, Cerenkov luminescent imaging, Tumor imaging, Passive targeting

## Abstract

**Background:**

Radioactive isotope-labeled gold nanomaterials have potential biomedical applications. Here, we report the synthesis and characterization of PEGylated crushed gold shell-radioactive iodide-124-labeled gold core nanoballs (PEG-^124^I-Au@AuCBs) for in vivo tumor imaging applications through combined positron emission tomography and Cerenkov luminescent imaging (PET/CLI).

**Results:**

PEG-^124^I-Au@AuCBs showed high stability and sensitivity in various pH solutions, serum, and in vivo conditions and were not toxic to tested cells. Combined PET/CLI clearly revealed tumor lesions at 1 h after injection of particles, and both signals remained visible in tumor lesions at 24 h, consistent with the biodistribution results.

**Conclusion:**

Taken together, the data provided strong evidence for the application of PEG-^124^I-Au@AuCBs as promising imaging agents in nuclear medicine imaging of various biological systems, particularly in cancer diagnosis.

**Electronic supplementary material:**

The online version of this article (10.1186/s12951-018-0366-x) contains supplementary material, which is available to authorized users.

## Background

In nanomedicine, gold nanomaterials have a number of unique merits, including easy surface modification via Au–S (gold–sulfur) bonds, efficient photothermal conversion, biocompatibility, and high stability in vivo [1–5]. Thus, these materials have been widely investigated for the development of radiotherapy [6, 7] or chemotherapy enhancers [8, 9], photothermal therapy (PTT) agents [10, 11], or photodynamic therapy (PDT) agents [12, 13].

Especially as a new imaging agents [6, 14–16], radiolabeled gold nanomaterials have attracted attention as nuclear medicine imaging probes owing to their high sensitivity and unlimited translational capability [17–19]. Xie et al. [20] prepared ^64^Cu-conjugated gold nanoshells for tumor diagnosis using DOTA-mediated chelation and PEGylation, and demonstrated these nanoshells can reveal tumor lesions for 3 h post-injection. Another study has shown that ^68^Ga-labeled gold glyconanoparticles using NOTA chelating agents, which are additionally functionalized with two related opioid peptides, act as targeting ligands enhancing blood brain barrier crossing [21]. Furthermore, Zhu et al. [22] demonstrated the efficient and rapid labeling of radioactive ^18^F onto gold nanoparticles (AuNPs) through maleimide/thiol click chemistry that shows the distribution of radioactive AuNPs in healthy rats. Although these studies have demonstrated successful tumor visualization and bio-distribution of these compounds in living subjects using positron emission tomography (PET), the loss of radioactive radioisotopes on AuNPs cannot be prevented because of the absence of rigid protective nanostructures, which leads to a decrease in the sensitivity and transchelation of radioactive isotopes to biological molecules and its sequential accumulation in nontarget organs, thereby increasing the risk of image misinterpretation. Therefore, the continued development of techniques is required to effectively protect the radioisotope embedded in AuNPs.

Recently, it has been documented that Cerenkov luminescence imaging (CLI) is a useful optical imaging modality that can be conducted using luminescent radionuclides, such as ^64^Cu, ^68^Ga, and ^124^I. The introduction of positron emitting radioisotopes into gold nanomaterials has facilitated CLI in several preclinical and clinical settings [23–26], overcoming the limitations of nuclear medicine imaging.

Well-designed gold nanostructures with optimal sizes can be accumulated in tumors through enhanced permeability and retention (EPR) effects [27, 28]. Furthermore, many attempts have been made to improve tumor targeting of imaging particles by changing the size or shape of nanostructures [29–33] or by modifying particle surfaces with hydrophilic polymers such as polyethylene glycol (PEG), which can interfere with aggregation and recognition by the reticuloendothelial system (RES) [34–36].

**Fig. 1:**
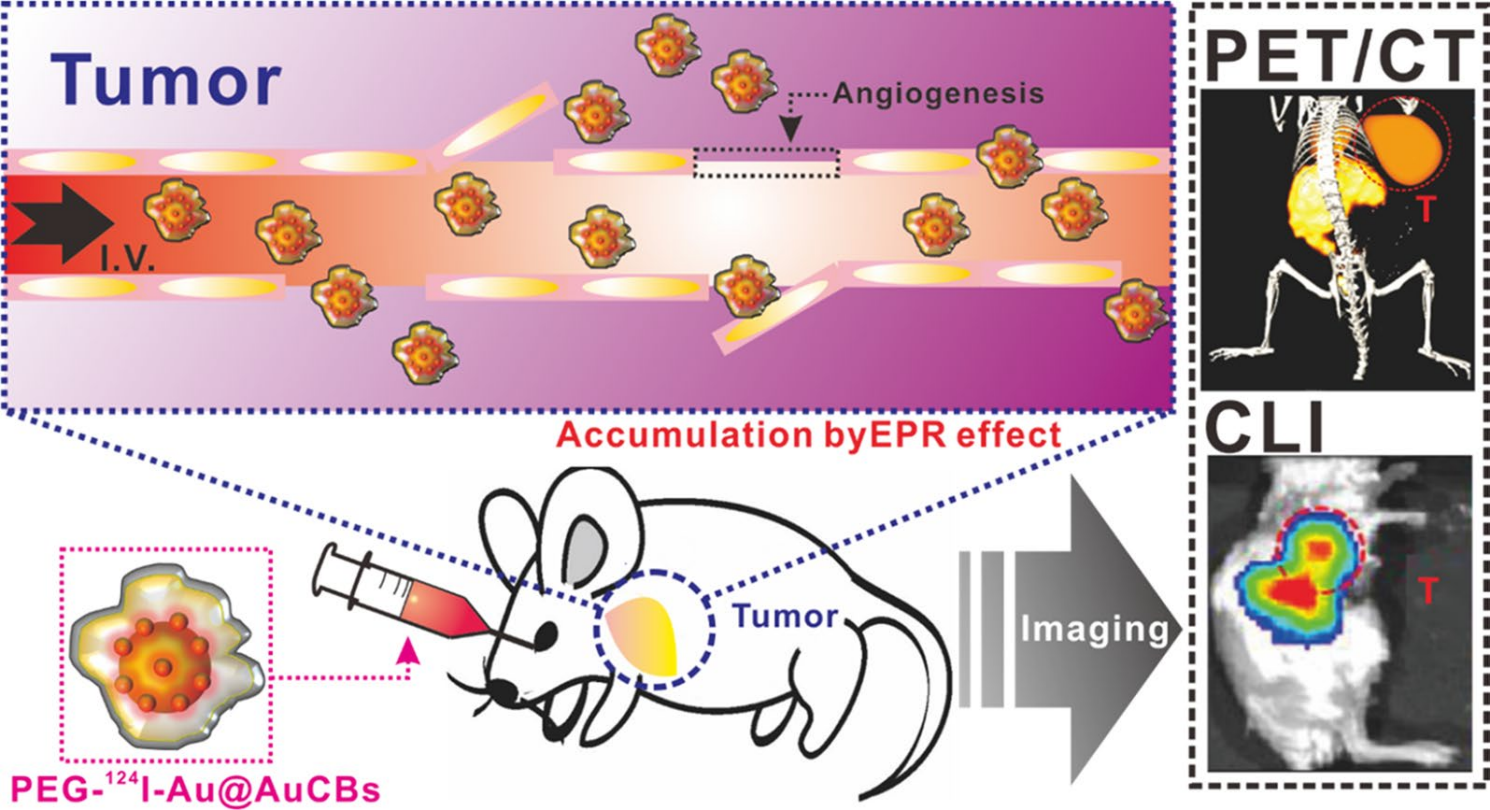
In vivo tumor imaging with PEG-^124^I-Au@AuCBs through combined PET and CLI. PEG-^124^I-Au@AuCBs were administered to mice through retro-orbital injection, and tumor imaging was conducted with combined PET/CLI. Red circles indicate the tumor region. *T* breast cancer tumor; *I.V* intravenous

We previously used tannic acid-coated gold nanoparticles (TA-AuNPs) to develop a novel synthetic approach for production of highly sensitive and stable radiolabeled gold nanoparticles with gold shells (^124^I-Au@AuNPs), and demonstrated the feasibility of this method for in vivo tracking of immunotherapeutic cells [37]. This method showed excellent sensitivity, stability, and biocompatibility, suggesting potential applications in disease diagnosis, particularly tumor diagnosis. On the basis of our previous findings, we postulated that ^124^I-Au@AuNPs could be useful for the detection of various types of cancers in living subjects. Thus, we explored whether ^124^I-Au@AuNPs could be targeted to tumor lesions by EPR effects following intravenous injection. To evaluate tumor targeting efficacy, ^124^I-Au@AuNPs were modified with PEG polymers (PEG-^124^I-Au@AuNPs) and administered into mice with breast cancer via tail vein injection, followed by biodistribution analysis at 24 h post-injection. As shown in Additional file 1: Figure S1, most injected radioactive particles accumulated in RES organs, including the liver and spleen [38], and radioactivity (0.015% ± 0.01% ID/g) was rarely found in breast tumors. This finding indicated that PEG-^124^I-Au@AuNPs were not suitable for in vivo tumor imaging via passive targeting owing to potential long-term toxicity [39, 40], thereby hampering their clinical use [41]. Therefore, these findings encouraged us to identify a new approach for enhancement of the tumor targeting ability of our novel imaging agents. Herein, through the introduction of changes in gold shell shape via modulation of pH concentrations, we developed novel PET/CLI imaging agents (^124^I-Au@AuCBs) consisting of radioactive ^124^I-labeled gold cores or crushed/PEGylated gold shells for visualization of tumor lesions in living mice. These materials were characterized, and the passive targeting capability of currently developed imaging agents were evaluated via EPR effects in a mouse model of breast cancer by biodistribution studies and combined PET/CLI imaging (Fig. 1).

## Methods

### Materials and imaging instruments

All chemical reagents, including HAuCl4, were purchased from Sigma-Aldrich (St. Louis, MO, USA). Tannic acid-capped gold nanoparticles (TA-AuNPs) were purchased from Ted Pella, Inc. (Redding, CA, USA), and Na124I was provided by KIRAMS (Seoul, South Korea) and Duchembio (Daegu, South Korea). PET/CT imaging was performed with a PET/CT scanner (LabPET8; Gamma Medica-Ideas, Waukesha, WI, USA). CLI was conducted with an IVIS Lumina III (PerkinElmer, USA).

### Animals and cells

Specific pathogen-free immunocompetent 6-week-old BALB/c mice were obtained fromSLC, Inc. (Shizuoka, Japan). All experimental procedures involving animals were conducted in strict accordance with the appropriate institutional guidelines for animal research. The protocol was approved by the Committee on the Ethics of Animal Experiments of the Kyungpook National University (Approval Number: KNU 2012-43).

Chinese hamster ovary (CHO) cells were grown in custom RPMI medium 1640 (Hyclone, Logan, UT, USA) supplemented with 10% fetal bovine serum (FBS; Hyclone) and 1% penicillin–streptomycin (Gibco, Grand Island, NY, USA). Murine dendritic (DC2.4) cells and breast cancer cells (4T1) were grown in RPMI medium 1640 supplemented with 10% FBS, 1% penicillin–streptomycin, 1.0 M β-mercaptoethanol (Sigma), and 1% minimum essential medium (MEM) nonessential amino acids (Gibco). Murine breast cancer 4T1 cells were grown in RPMI medium 1640 supplemented containing 10% FBS and 1% penicillin–streptomycin (Gibco).

### Preparation of radioactive gold core nanoparticles (^124^I-AuNPs)

Iodination was performed by adding 100 μL of 222 MBq Na^124^I into 1.0 mL of 1.0 nM AuNPs in the presence of 90 μL of 3 mg/mL chloramine-T and 100 μL of 1% SDS to produce ^124^I-AuNPs. The reaction, which was monitored by radioactive thin layer chromatography (radio-TLC: eluent, acetone), was completed within 15 min. After the reaction, the solution was centrifuged to remove free Na^124^I, and precipitates were redispersed in distilled water.

### Calculation of ^124^I density per AuNPs (^124^I-AuNPs)

AuNPs concentration was determined by measuring the absorption at 520 nm using a UV–VIS spectrometer. The following parameters were used:

1 $${\text{Concentration}}\;{\text{of}}\;^{124} {\text{I}} = 0.1\;{\text{Ci}}/{\text{L}}\,(0.001\;{\text{Ci}}/{\text{mL}})$$

2 $${\text{Specific activity of}}\,^{ 1 2 4} {\text{I}} = 1 7 {\text{ Ci}}/{\text{mg}} = 1 7 ,000{\text{ Ci}}/{\text{g}}$$

3 $${\text{Molecular weight of}}\,^{ 1 2 4} {\text{I}} = 1 2 5 {\text{ g}}/{\text{mol}}$$

4 $${\text{The number of moles in 2}}0{\text{ nm AuNPs}} = 1.0 9 { } \times { 1}0^{ - 1 2}$$

5 $${\text{Absorbance }} = { 1}.0,{\text{ extinction }} = { 9}. 2 1 { } \times { 1}0^{ 8} ;{\text{ absorbance}}/{\text{extinction }} = {\text{ number of 2}}0{\text{ nm AuNPs }}\left( {{\text{OD52}}0} \right)$$

6 $${\text{AuNP volume}}{:}\, 1.0{\text{ mL}}$$

#### Calculation of the number of ^124^I moles

From (2) and (3):

7 $$1 7,000{\text{ Ci}}/{\text{g }} \times {\text{ 125 g}}/{\text{mol}} = 2,1 2 5,000{\text{ Ci}}/{\text{mol}} = 2 1 2 5 {\text{ Ci}}/{\text{mmol}}$$

From (1) and (7):

8 $$0.000 1 {\text{ (Ci}}/{\text{mL}})/ 2 1 2 5 {\text{ Ci}}/{\text{mmol}} = 0.0 4 5 9 7\, { }\upmu{\text{mol}}$$

9 $$\begin{aligned} {\text{The number of}}\,^{ 1 2 4} {\text{I moles}} & = 0.0 4 5 9 7 \,{ }\upmu{\text{mol }} \times \, 0. 2 5 { }\left( { 5 {\text{mCi}}} \right) \, \\ & = \, 0.0 1 1 4 9 2 5\, { }\upmu{\text{mol}} \\ & = { 1}. 1 5 { } \times { 1}0^{ - 8} \,{\text{mol}} \\ \end{aligned}$$

#### Calculation of the number of AuNP moles

From (5) and (6):

10 $${\text{Absorbance}}/{\text{extinction }} \times {\text{ volume}} = 1.0/ \, \left( { 9. 2 1 { } \times { 1}0^{ 8} } \right) \, \times { 1}.0{\text{ mL = 1}}.0 9 { } \times { 1}0^{ - 1 2}\, {\text{mol }}$$

#### Number of ^124^I per AuNP

From (9) and (10), $${\text{the number of}}^{ 1 2 4} {\text{I}}/{\text{moles per }}20\text{-}{\text{nm AuNP}} = 1. 1 5 { } \times { 1}0^{ - 8}\, {\text{mol}}/ 1.0 9 { } \times { 1}0^{ - 1 2}\, {\text{mol}} = 10, 5 7 5$$

### Preparation of PEGylated crushed gold shell-radioiodine-124-labeled gold core nanoballs (^124^I-Au@AuCBs)

To generate a crushed gold shell on ^124^I-AuNPs, 1.0 mL of ^124^I-AuNPs (1.0 nM) was mixed with 500 μL of 1.0% (w/v) poly(*N*-vinyl-2-pyrrolidone) (MW, 40 kDa) and 100 μL of 100 mM phosphate buffer (pH 12.0). The solution was mixed with 434 μL of hydroxylamine hydrochloride (10 mM) and 434 μL of HAuCl_4_ (5 mM), gently vortexed for 30 min at room temperature, and centrifuged at 6500 rpm for 15 min twice. The supernatant was resuspended in 1.0 mL distilled water. To prepare 1 mL of PEGylated ^124^I-Au@AuCBs, a freshly prepared ^124^I-Au@AuCBs solution (1.0 mL, 1.0 nM) was reacted with mPEG-SH (MW: 5000 Da, 10^4^ equivalents, 30 μL, 1 mg/mL) for 24 h at room temperature and then purified via centrifugation (6500 rpm, 15 min). The supernatant was removed and, particles were redispersed in phosphate buffer (1.0 mL, pH 7.4).

### Characterization of nanoparticles

UV–visible spectroscopy was conducted using a Cary 60 UV–Vis spectrophotometer (Agilent Technologies, Santa Clara, CA, USA), and X-ray photoelectron spectroscopy was performed using a Quantera SXM instrument (ULVAC-PHI; Chiasaki, Kanagawa, Japan). Transmission electron microscopy and energy dispersive X-ray mapping were performed using an FEI Tecnai F20 transmission electron microscope (FEI Company, Eindhoven, the Netherlands). X-ray photoelectron spectroscopy (XPS) and crystallography of the products were carried out using a PANalytical X-ray diffractometer. The hydrodynamic sizes of the nanoparticles were measured using a ζ-potential and particle size analyzer (ELS-Z, Otsuka, Japan). Powder XRD patterns were recorded on a Philips X’Pert PRO SUPER X-ray diffract meter system with CuKα radiation (λ = 1.542 Å, 40 kV, 30 mA) source. Fourier transform infrared (FT-IR) spectra were recorded on a Perkin-Elmer spectrometer in the range between 4000 and 400 cm^−1^.

### Sensitivity tests

Changes in both PET and CLI signals with varying concentrations of PEG-^124^I-Au@AuCBs (1.0 × 10^−13^ to 1.0 × 10^−10^) were examined using a PET system and an IVIS Lumina III (Perkin Elmer).

### Stability tests in various pH solutions and serums

One hundred microliters of 1.0 nM^124^I-Au@AuCBs was incubated with 900 μL of various pH solutions and serum at 37 °C. The released radionuclide was quantified by TLC using an AR-2000 scanner (Bioscan, Washington, DC, USA).

### Cell proliferation assays

Cell proliferation assays were performed using a Cell Counting Kit (CCK-8; Dojindo Laboratories, Tokyo, Japan) and CellTiter-Glo^®^ Luminescent Cell Viability Assay (Promega, Madison, USA). CHO, DC2.4, and 4T1 cells were seeded at 1 × 10^4^ cells/well in 96-well plates. Either ten microliters of CCK-8 solution or CellTiter-Glo reagents was added to each well at 24 and 48 h after incubation with PEG-^124^I-Au@AuCBs, and the plates were then incubated 37 °C for 1 h. Absorbance (at 450 nm) and luminescence was measured using a microplate reader (BMG Labtech, Offenburg, Germany).

### Apoptosis analysis

CHO, DC2.4, and 4T1 cells were plated in 6-well plates. Cells were collected and stained with a solution of fluorescein isothiocyanate (FITC)-conjugated annexin V and propidium iodide (BD Science) after 24 and 48 h of incubation with PEG-^124^I-Au@AuCBs. Flow cytometric analysis was performed using a BD Accuri C6 Flow cytometry (BD Biosicences).

### In vivo imaging of combined PET and CLI

Breast cancer cell line (4T1) tumors were established by subcutaneous injection of 1 × 10^6^ cells to right upper flank. When tumor volume is detectable by inspection and palpation, in vivo study was done.

4T1 tumor-bearing mice (n = 5) received PEG-^124^I-Au@AuCBs (Na^124^I:1.48 MBq, AuCBs:16 pM) via retro-orbital injection, and combined PET/CT and CL images were acquired at 1, 6, and 24 h postinjection.

For the PET/CT study, a 20-min (tumor imaging) scan was performed using the Triumph II PET/CT system (LabPET8; Gamma Medica-ideas, Wausha, WI, USA). The PET imaging system has the following characteristics: ring diameter, 162 mm; FOV, 60 mm; crystals, 3072; spatial resolution, 1.35 mm FWHM FOV; and noise-equivalent counts, 37 kcps at 245 MBq (250–650 keV). CT scans were performed with an X-ray detector (fly acquisition; number of projections: 512; binning setting: 2 × 2; frame number: 1; X-ray tube voltage 75 kVp; focal spot size 50 μm; magnification factor 1.5; matrix size 512) immediately following the acquisition of PET images. PET images were reconstructed using 3D-OSEM iterative image reconstruction. CT images were reconstructed using filtered back-projections. All mice were anesthetized using 1–2% isoflurane gas during imaging. PET images were co-registered with anatomical CT images using three-dimensional image visualization and analysis software (VIVID; Gamma Medica-ideas, Northridge, CA, USA). To measure the uptake (counts) for the volumes of interest (VOIs), each image was manually segmented from co-registered CT images using VIVID, and the radioactivity in the ROI was determined.

For in vivo CLI, images were acquired using an IVIS Lumina III imaging system (PerkinElmer). All luminescence images were measured from a bioluminescence channel with no excitation light or emission filters using IVIS Lumina III. The scan time was varied from 5 s to 5 min depending on the intensity of the emitted luminescence signal. The luminescence image was thresholded to maximize the visualization of the region of interest and to minimize background. Grayscale photographic images and bioluminescent color images were superimposed using LIVINGIMAGE version 2.12 (PerkinElmer) and IGOR Image Analysis FX software (WaveMetrics, Lake Oswego, OR, USA). CLI signals were expressed in units of photons per cm^2^ per second per steradian (P cm^−2^ s^−1^ sr^−1^).

### Biodistribution study

Mice (n = 5) were sacrificed at 1, 6, and 24 h post-injection of PEG-^124^I-Au@AuCBs. Tissues were weighed and counted on a gamma counter. Uptake in each tissue was expressed as the percentage injected dose per gram of tissue (%ID g^−1^).

### Ex-vivo PET and CLI

At 24 h postinjection of particles, mice were killed. Organs of interest, including tumors, were excised and placed in 6-well plates, followed by ex vivo PET/CT and CLI.

### Histological examination

4T1 tumors were excised, fixed in formalin, embedded in paraffin, sectioned, stained with hematoxylin and eosin (H&E), and analyzed by light microscopy. The presence of gold particles in tumor lesions was determined by a histological specialist.

### Statistical analysis

All data are expressed as means ± standard deviations, from at least three repeated experiments. Statistical significance was determined using unpaired Student’s t tests with Graph Pad Prism version 5 statistical software (GraphPad Software, Inc.) Differences with *p* values of less than 0.05 were considered statistically significant.

## Results and discussion

### Characterization of PEGylated crushed gold shell-radiolabeled core nanoballs (PEG-^124^I-Au@AuCBs)

**Fig. 2:**
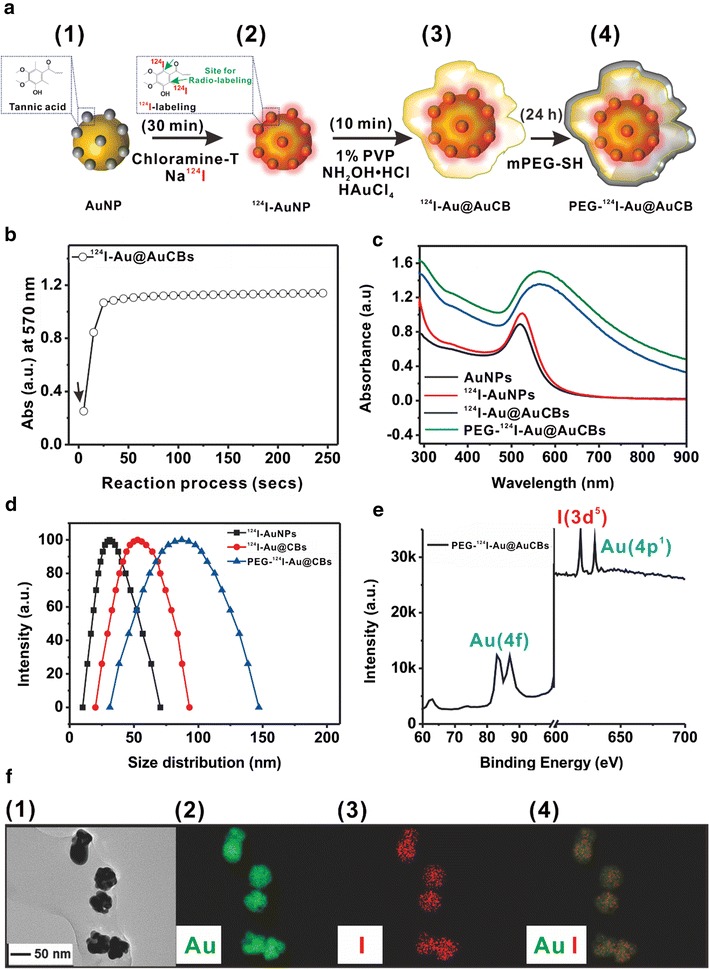
Synthesis of PEG-^124^I-Au@AuCBs. **a** Tannic acid-functionalized gold nanoparticles were first labeled with ^124^I (1, 2), followed by gold shell formation (3) and PEGylation (4). **b** Reaction rate of Au-shell coverage on ^124^I-AuNPs. **c** UV–visible spectroscopy analysis of AuNPs, ^124^I-AuNPs, ^124^I-Au@AuCBs, and PEG-^124^I-Au@AuCBs. **d** Dynamic light scattering analysis of AuNPs, ^124^I-AuNPs, ^124^I-Au@AuCBs, and PEG-^124^I-Au@AuCBs. **e** X-ray photon electron spectroscopy of PEG-^124^I-Au@AuCBs. **f** (1) TEM image of PEG-^124^I-Au@AuCBs and energy dispersive X-ray (EDX)-based elemental mapping of (2) Au and (3) I; a merged image is also shown (4)

The shape and size of nanoparticles have been reported to be important factors for enhanced blood circulation and improved disease-targeting efficacy through minimizing nonspecific accumulation in RES organs. Thus, we attempted to produce effective tumor imaging probes via a passive approach by modifying the shape of gold shell nanostructures in PEG-^124^I-Au@AuNPs. For production of effective tumor imaging agents, radioactive iodine-labeled gold core nanoparticles (^124^I-AuNPs) were first produced (Fig. 2a (1) and (2)). TA-AuNPs have multiple functional sites that can be labeled with numerous radioactive iodides [42], and ^124^I has been extensively investigated for PET imaging in preclinical and clinical settings [43]. Furthermore, many reports have demonstrated that ^124^I is a useful radionuclide for CLI [24].

**Fig. 3:**
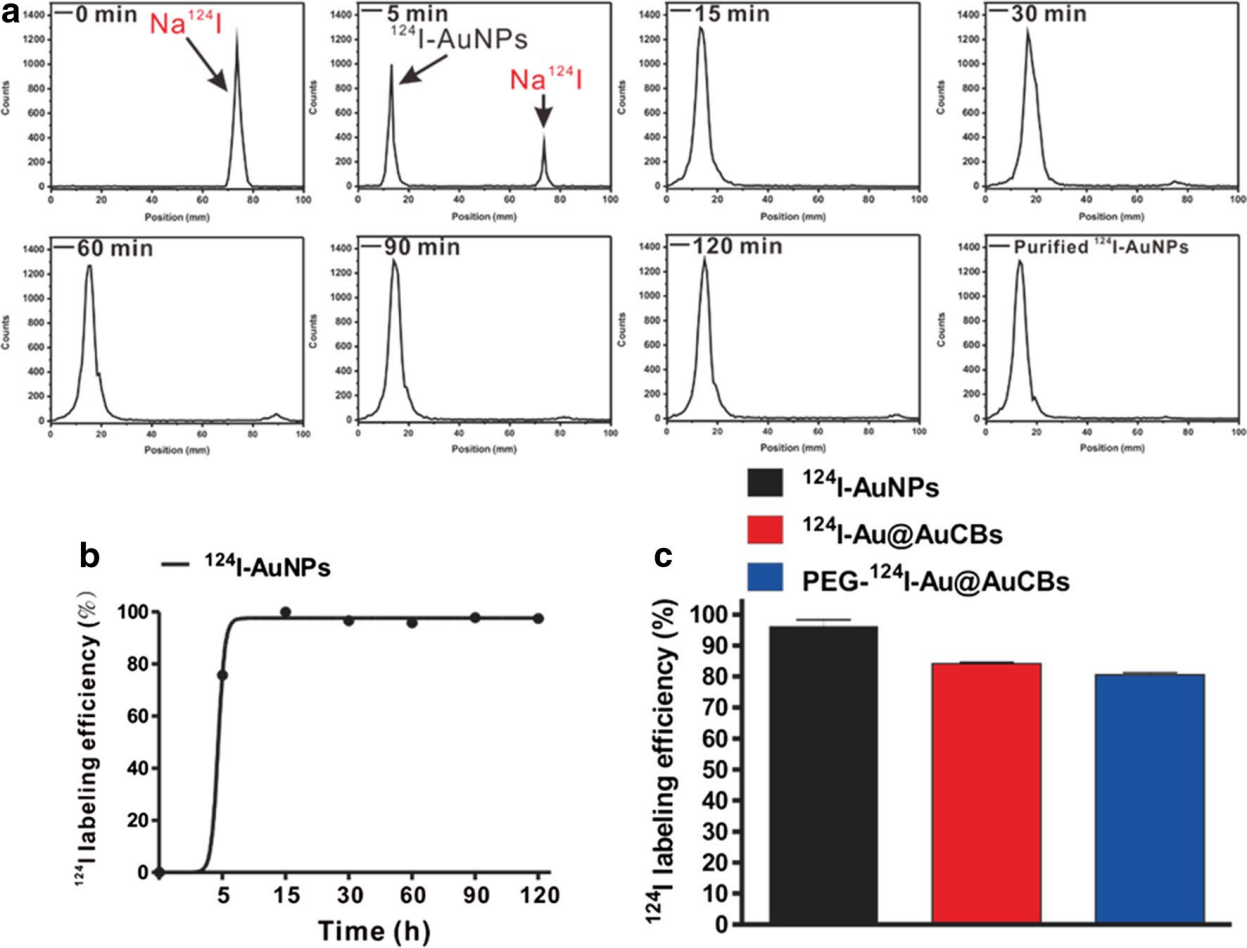
Radio-labeling characterization of PEG-^124^I-Au@AuCBs. **a** Time-dependent radiochromatography of TLC to monitor the radiolabeling reaction. **b** Kinetic analysis of the ^124^I labeling reaction. **c** Radioiodine labeling efficiency of ^124^I-AuNPs, ^124^I-Au@AuCBs, and PEG-^124^I-Au@AuCBs. Data are presented as mean ± standard deviation (SD)

**Fig. 4:**
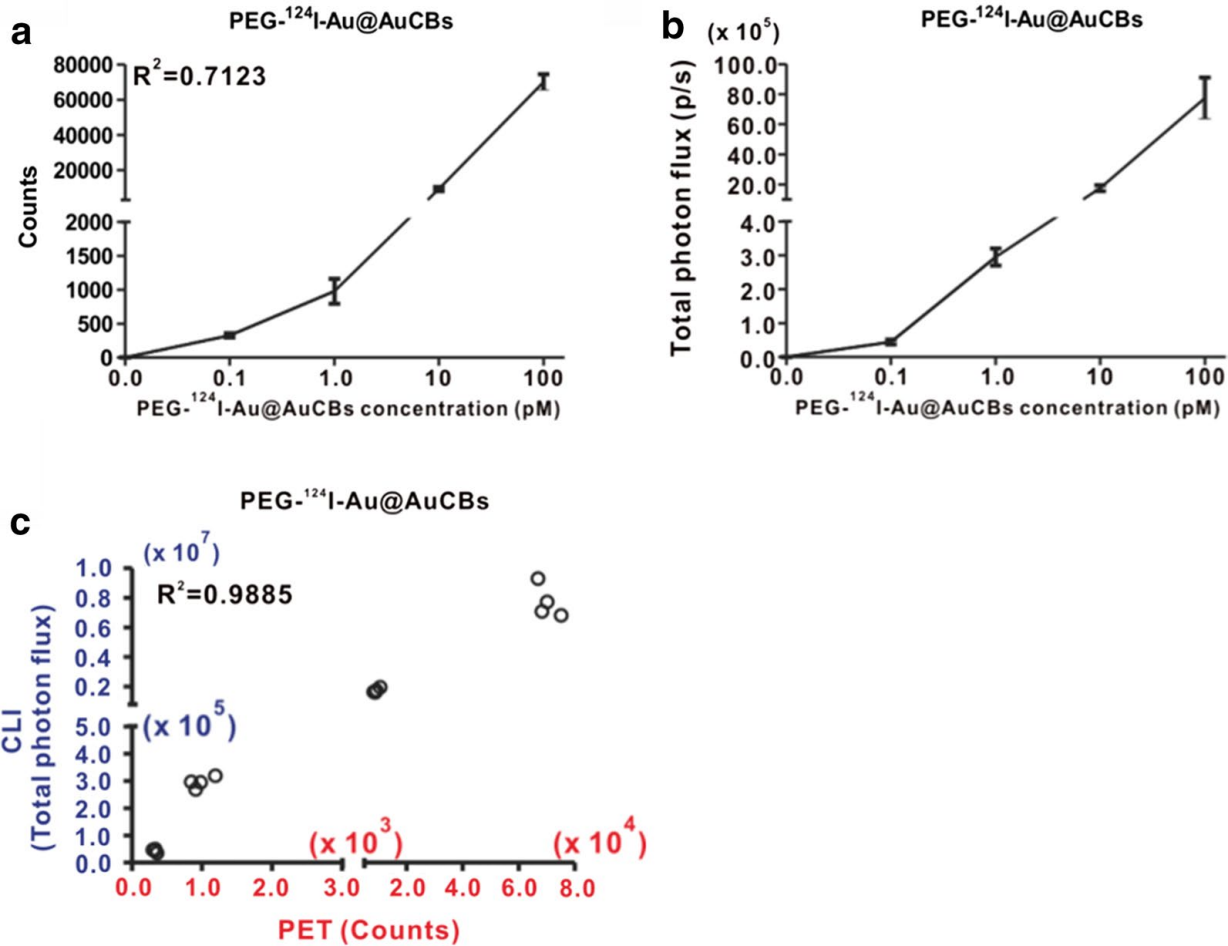
Sensitivity of PEG-^124^I-Au@AuCBs (**a**, **b**). Quantification of radioactivity and Cerenkov luminescence signal of PEG-^124^I-Au@AuCBs as a function of concentration. **c** Correlation between PET signal and CLI signal of PEG-^124^I-Au@AuCBs. Multiwell plates containing solutions and PEG-^124^I-Au@AuCB particles were exposed to PET instruments and an IVIS imaging system, and radioactivity and CLI signals were quantified. Data are presented as mean ± standard deviation (SD)

Thus, TA-functionalized gold nanoparticles were reacted with ^124^I by simple incubation and stirring at room temperature, and radiolabeling progression was monitored with a radio-TLC scanner. As illustrated in Fig. 3, complete radiolabeling of TA-AuNPs was observed within 15 min, with a final radiochemical yield of 98% (Fig. 3c, black bar). Perrault et al. [44] reported that rod-shaped nanomaterials have much higher blood circulation half-lives than spherical nanomaterials. Moreover, Lee et al. [45] reported the successful control of gold shell nanostructures such as a star-shaped shell with irregular nanogap and intra-nanogap distances by pH and NaCl concentrations. Accordingly, we attempted to modulate the shape of gold shell nanostructures through modulations of pH to protect the release of radioiodine from gold core nanoparticles by various biological factors in living subjects and effectively enhance passive tumor targeting efficiency. When additional gold shells were reacted onto ^124^I-AuNPs in solution at pH 12, we observed crushed gold shell formation as early as 20 s after the start of the reaction, with a maximum at 50 s (Fig. 2b). The radiochemical yield of ^124^I-Au@AuCBs was high (84%; Na^124^I 0.094 GBq, AuCBs 1 nM; Fig. 3c, red bar). To improve the blood circulation of ^124^I-Au@AuCBs in vivo, these particles were further functionalized through 10^4^ molar excess of PEGylation [MW: 5000; PEG-^124^I-Au@AuCBs; Fig. 2a, (4)]. The final radiochemical yield of PEG-^124^I-Au@AuCBs was 80% (Na^124^I: 0.089 GBq, mole: 1 nM; Fig. 3c, blue bar). Ultraviolet (UV)-visible spectroscopy of PEG-^124^I-Au@AuCBs exhibited an identical wavelength to that of intermediate gold nanoparticles, indicating the absence of nanostructure aggregation during the reaction (Fig. 2c). Dynamic light scattering analysis revealed a hydrodynamic radius of 31.1 ± 1.3, 52.8 ± 2.1, and 87.1 ± 3.5 for ^124^I-AuNPs, ^124^I-Au@AuCBs, and PEG-^124^I-Au@AuCBs, respectively (Fig. 2d). X-ray photoelectron spectroscopy revealed the presence of Au and iodine in PEG-^124^I-Au@AuCBs (Fig. 2e). Furthermore, PEG-^124^I-Au@AuCBs had a bumpy surface morphology, as revealed by high-resolution transmission electron microscopy (HR-TEM; Fig. 2f (1)). Subsequently, the distribution of iodine was observed around the AuCBs (Fig. 2f, (2–4)) via X-ray energy dispersive mapping analysis. Zeta-potential (ζ-potential) analysis demonstrated that the surface charges of respective particles were − 46.13 ± 4.6, − 53.20 ± 0.8, − 32.97 ± 1.5, and − 0.62 ± 0.3 mV for AuNPs, ^124^I-AuNPs, ^124^I-Au@AuCBs, and PEG-^124^I-Au@AuCBs, respectively (Additional file 1: Figure S2b). Fourier transform infrared spectroscopy (FT-IR) analysis confirmed the presence of carbon, hydrogen, and oxygen bonding changes peaks in gold nanoparticles (Additional file 1: Figure S2a). X-ray diffraction (XRD) analysis also clearly showed the gold nanostructure, revealing unique Au reflections at 111, 200, 220, and 311° in the XRD spectra of PEG-^124^I-Au@AuCBs (Additional file 1: Figure S2c). HR-TEM analysis showed that the Au–Au lattice sizes ranged from 0.21 to 0.25 nm (Additional file 1: Figure S3).

### In vitro evaluation of the sensitivity, stability, and cytotoxicity of PEG-^124^I-Au@AuCBs

The sensitivity and stability of newly developed imaging agents are essential for selective detection and follow-up in various diseases [18]. Thus, we examined the sensitivity of PEG-^124^I-Au@AuCBs with combined PET/CLI following serial dilution of the particles. Radioactivity and CLI signals increased in a dose-dependent manner, with good linearity between both imaging signals and particles (Fig. 4a–c, Additional file 1: Figure S4; R^2^ = 0.72 for PET, R^2^ = 0.77 for CLI). Radioactivity and CLI signals were detected as low as 0.1 pM, indicating high sensitivity. Furthermore, we observed a good correlation between PET signals and CLI signals (R^2^ = 0.98 for PET and CLI; Fig. 4d).

**Fig. 5:**
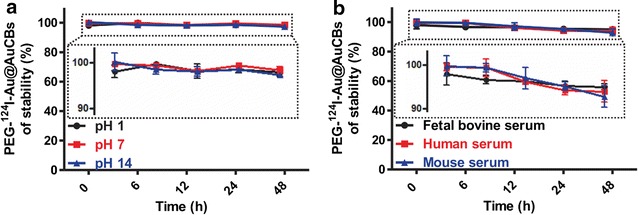
Stability of PEG-^124^I-Au@AuCBs in various pH solutions and serum. **a** Time-dependent pH stability of PEG-^124^I-Au@AuCBs at pH 1, 7, and 14. **b** Time-dependent serum stability of PEG-^124^I-Au@AuCBs in fetal bovine serum, human serum, and mouse serum. Data are presented as mean ± standard deviation (SD)

In addition, we tested the stability of PEG-^124^I-Au@AuCBs in solutions with different pH and in serum. Notably, PEG-^124^I-Au@AuCBs exhibited high stability (more than 90%) in solutions ranging from pH 1 to 14 (Fig. 5a, b, Additional file 1: Figures S5, S6). In various types of serum, PEG-^124^I-Au@AuCBs also showed strong stability over 48 h (Fig. 5d–f, Additional file 1: Figure S5).

Next, we determined the biocompatibility of our PEG-^124^I-Au@AuCBs by examining the cytotoxicity of these agents in various cell types. As shown in Additional file 1: Figure S7, no differences in cell viability were observed in unlabeled and labeled groups at the tested concentrations of PEG-^124^I-Au@AuCBs. Consistent with the results of cell proliferation assays, there were no differences in the level of apoptosis between unlabeled and labeled groups at all tested concentrations of PEG-^124^I-Au@AuCBs (Additional file 1: Figure S8). These results suggest that PEG-^124^I-Au@AuCBs had essential features of in vivo bioimaging probes.

### In vivo detection of breast cancer with dual PET and CLI imaging

**Fig. 6:**
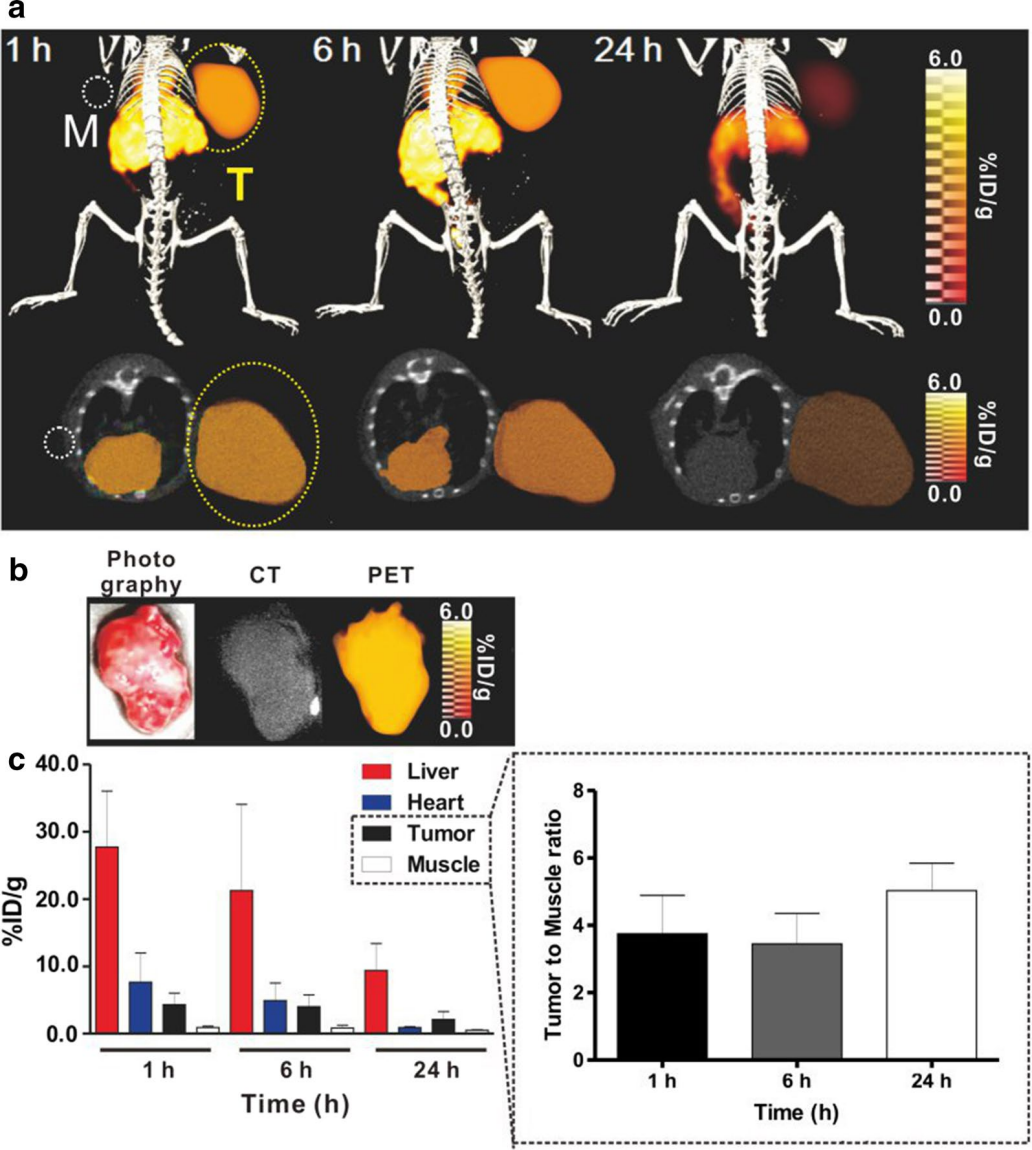
In vivo PET imaging of breast cancer with PEG-^124^I-Au@AuCBs. **a** PET/CT images showing tumor lesions in mice with breast cancer following injection of PEG-^124^I-Au@AuCBs. Upper: 3D-PET/CT images; lower: axial PET/CT images. White and yellow circles indicate muscle and tumor lesions, respectively. **b** PET image of an excised tumor. **c** Region of interest (ROI) analysis of radioactivity in the liver, heart, muscle, and tumor. The stippled black box shows the uptake ratio of tumor to muscle. Five mice were used for all time points. Data are presented as mean ± standard deviation (SD)

**Fig. 7:**
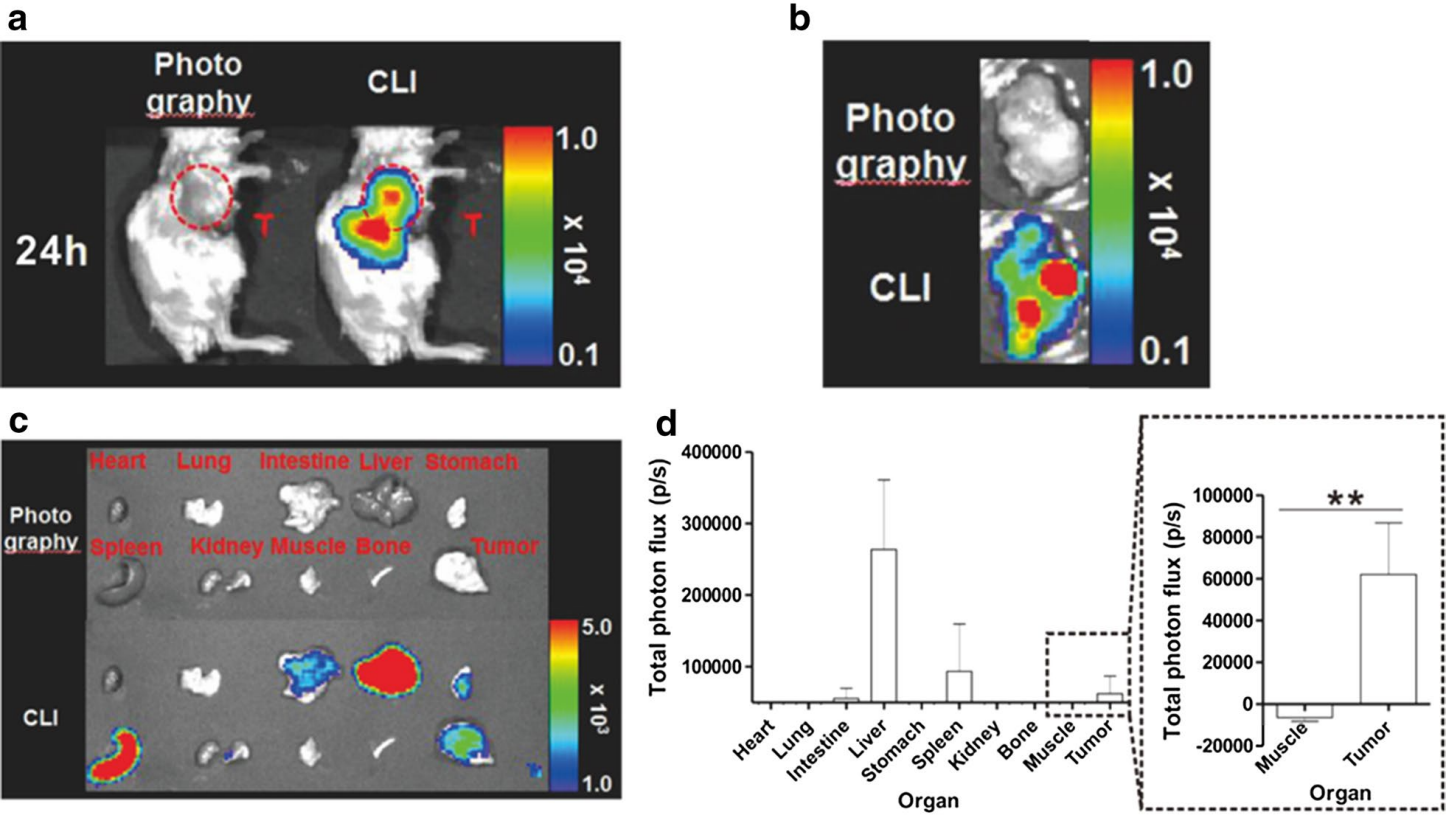
In vivo CLI of breast cancer with PEG-^124^I-Au@AuCBs. **a** In vivo CLI for detection of breast cancer with ^124^I-Au@AuCBs at 24 h post-injection. Left: photograph, right: CLI. **b** Ex vivo CLI of an excised tumor. **c** CLI of other excised organs and the tumor. **d** Region of interest (ROI) analysis of luminescence activity in organs shown in (**c**). The stippled black box shows a magnified image. Five mice were used for each time point (1, 6, and 24 h post-injection). Data are presented as mean ± standard deviation (SD). **p < 0.01

The feasibility of PEG-^124^I-Au@AuCBs for in vivo detection of tumor lesions was then assessed in a mouse model of breast cancer. As shown in Additional file 1: Figure S9, in vivo experiments were carried out, and PET/CT imaging showed the distinct uptake of PEG-^124^I-Au@AuCBs at tumor sites as early as 1 h post-injection, with a strong radioactive signal detectable at 24 h (Fig. 6a, c), consistent with the biodistribution examination (Additional file 1: Figure S10). The uptake value of particles in the tumor region (% injected dose per gram (%ID/g)) were 5.38 ± 3.57, 3.42 ± 2.15, and 1.81 ± 1.30 at 1, 6, and 24 h post-injection, respectively. Consistent with PET/CT scanning, in vivo CLI clearly visualized the accumulation of PEG-^124^I-Au@AuCBs in breast tumor lesions at 24 h after particle injection (Fig. 7a), a finding which was consistent with ex vivo CLI of excised organs (Fig. 7c). Accordingly, ex vivo imaging of combined PET/CLI clearly showed intensive signals in tumor lesions (Figs. 6b and 7b, Additional file 1: Figure S11). PET and CLI imaging of breast cancer revealed great linearity between the PET and CLI signals at 24 h (R^2^ = 0.85, Additional file 1: Figure S12). As shown in Figs. 6 and 7, histological analysis demonstrated the accumulation of black nanocrushed ball-laden angiogenic vascular wall regions of tumors (Additional file 1: Figure S13), indicating the feasibility of applying PEG-^124^I-Au@AuCBs as in vivo tumor imaging agents. Future efforts are needed to reduce RES accumulation and enhance passive tumor targeting efficiency through the introduction of various coating systems, including hyaluronic acid, human chorionic gonadotrophin, and antibodies.

## Conclusions

In this study, we described the synthesis of PEG-^124^I-Au@AuCBs as novel oncological radionuclide biomedical imaging agents for in vivo tumor detection via passive tumor targeting. PEG-^124^I-Au@AuCBs showed good sensitivity, stability, and biocompatibility under various biological conditions. Importantly, PEG-^124^I-Au@AuCBs facilitated rapid detection of breast carcinoma with a low dose of imaging agents through a passive targeting system with combined PET/CLI. Thus, our imaging agents may hold great promise for applications as inorganic nanomedicines in clinical practice. Although PEG-^124^I-Au@AuCBs exhibit remarkable potential as tumor detection agents, major hurdles such as significant pathophysiological heterogeneity and the PEG dilemma, need to be overcome prior to their adoption in clinical practices via the RES system.

## Additional file

**Additional file 1.** Additional Figures S1–S13.

## Abbreviations

AuNP: gold nanoparticle
Au–S: gold–sulfur
CLI: Cerenkov luminescence imaging
EPR: enhanced permeability and retention
FT-IR: Fourier transform infrared spectroscopy
HR-TEM: high-resolution transmission electron microscopy
PDT: photodynamic therapy
PET: positron emission tomography
PTT: photothermal therapy
PEG: polyethylene glycol
RES: reticuloendothelial system
ROI: region of interest
SD: standard deviation
TA-AuNP: tannic acid-coated gold nanoparticle
^124^I-AuNP: iodide-labeled gold core nanoparticle
^124^I-Au@AuNP: radiolabeled gold nanoparticle with gold shell
PEG-^124^I-Au@AuNP: ^124^I-Au@AuNP modified with PEG polymers
UV: ultraviolet
XRD: X-ray diffraction
