# Supplementary material for: PEGylated crushed gold shell-radiolabeled core nanoballs for in vivo tumor imaging with dual positron emission tomography and Cerenkov luminescent imaging
Source: J Nanobiotechnology. 2018 Apr 18;16:41. doi: 10.1186/s12951-018-0366-x (PMC5907375; doi:10.1186/s12951-018-0366-x)
Supplement: Supplementary file 1 — Additional file 1. Additional Figures S1–S13. [file 12951_2018_366_MOESM1_ESM.docx]

**Additional Materials**

**PEGylated Crushed Gold Shell-radiolabeled Core Nanoballs for *In Vivo* Tumor Imaging with Dual Positron Emission Tomography and Cerenkov Luminescent Imaging**

Sang Bong Lee ^1,6^, Dinesh Kumar ^2^, Yinghua Li ^3^, In-Kyu Lee ^1,4^, Sung Jin Cho ^5^, Sang Kyoon Kim ^8^, Sang-Woo Lee ^1,6^, Shin Young Jeong ^6^, Jaetae Lee ^6,7*^ and Yong Hyun Jeon ^1,8^*

^1^Department of Nuclear Medicine, Kyungpook National University Hospital, Daegu, 702-210, South Korea

^1^Leading-edge Research Center for Drug Discovery and Development for Diabetes and Metabolic Disease, Kyungpook National University Hospital, Daegu, 702-210, South Korea

^2^Department of Bionanosystem Engineering, Graduate School, Chonbuk National University, Jeonju, Republic of Korea

^3^Department of Pathology, Chemon Co. Ltd., 240, Nampyeong-Ro, Yangji-Myeon, Cheoin-Gu, Yongin-Si, Gyeonggi-Do 17162, Republic of Korea

^4^Department of Internal Medicine, Kyungpook National University School of Medicine, Deagu 700-721, South Korea

^5^New Drug Development Center, Daegu-Gyeongbuk Medical Innovation Foundation, Daegu, South Korea

^6^Department of Nuclear medicine, Kyungpook National University School of Medicine, Daegu 700-721, South Korea

^7^Daegu-Gyeongbuk Medical Innovation Foundation, Daegu 360-4, South Korea

^8^Laboratory Animal Center, Daegu-Gyeongbuk Medical Innovation Foundation, Daegu 360-4, South Korea

***Correspondence to**:

Yong Hyun Jeon, PhD

Laboratory Animal Center, Daegu-Gyeongbuk Medical Innovation Foundation, Daegu 360-4, South Korea

H.P: 82-10-2455-6046

Work: 82-53-790-5726

E-mail: jeon9014@gmail.com

Jaetae Lee, MD, PhD

Department of Nuclear Medicine

Kyungpook National University School of Medicine

50 Samduk-dong 2-ga, Chung Gu, Daegu700-721, South Korea

Tel: 82-53-200-5577; Fax: 82-53-422-0864

E-mail: jaetae@knu.ac.kr

**
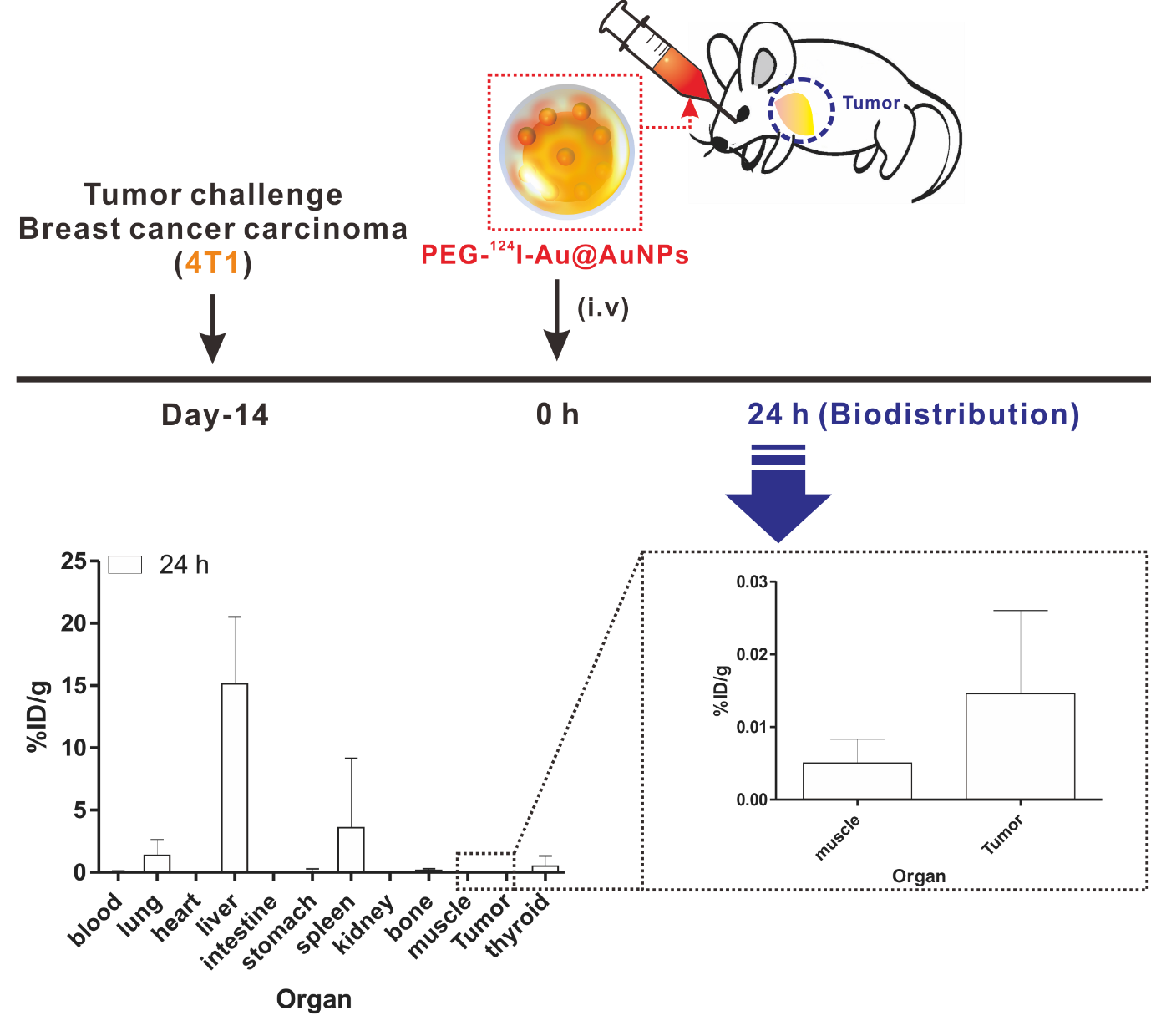
**

**Fig. S1.** Biodistribution of PEGylated complete shell gold nanoparticles (PEG-^124^I-Au@AuNPs) in whole organs after retro-orbital injection. The black box shows the magnified image. %ID/g, percentage injected dose per gram.

**
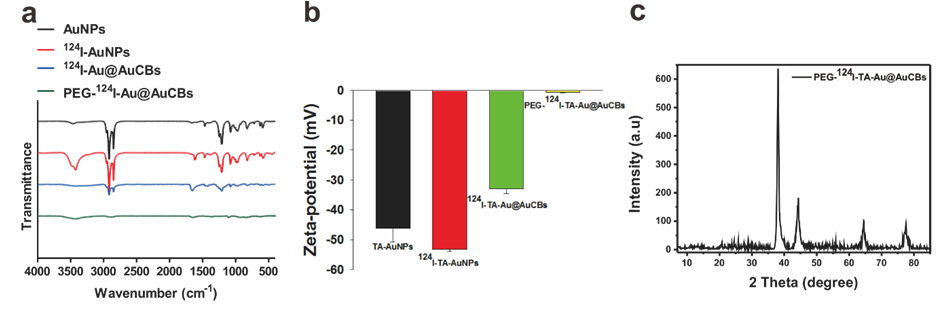
**

**Fig. S2.** Characterization of PEG-^124^I-Au@AuCBs. (**a**)FT-IR of AuNPs, ^124^I-AuNPs, ^124^I-Au@AuCBs, and PEG-^124^I-Au@AuCBs.The FT-IR spectrum analysis showed peaks at 1213 cm^-1^ for CH_2_, 1651 cm^-1^ for C=O stretching, 2858 cm^-1^ for C-H stretching, and 3408 cm^-1^ for O-H stretching. The FT-IR spectrum of AuNP products was also the same as that of the respective gold nanoparticle chemical reaction product.(**b**)Zeta-potentials of AuNPs, ^124^I-AuNPs, ^124^I-Au@AuCBs, and PEG-^124^I-Au@AuCBs.The introduction of functional groups, such as NH_2_ and COOH would lead to the change of surface charges of AuNPs (- 46.13 mV) after each reaction step. Thus, the analysis of zeta-potentials was conducted to further examine the chemical reaction of various functional groups to gold nanoparticles. In results, the values of zeta-potential (ζ-potential) revealed are -53.20 mV, -32.97, and -0.62 mV in ^124^I-AuNPs, ^124^I-Au@AuCBs, and PEG-^124^I-Au@AuCBs. These results suggest that various items/groups were successfully reacted to gold nanostructures. (**c**) XRD spectrum data of PEG-^124^I-Au@AuCBs.

**
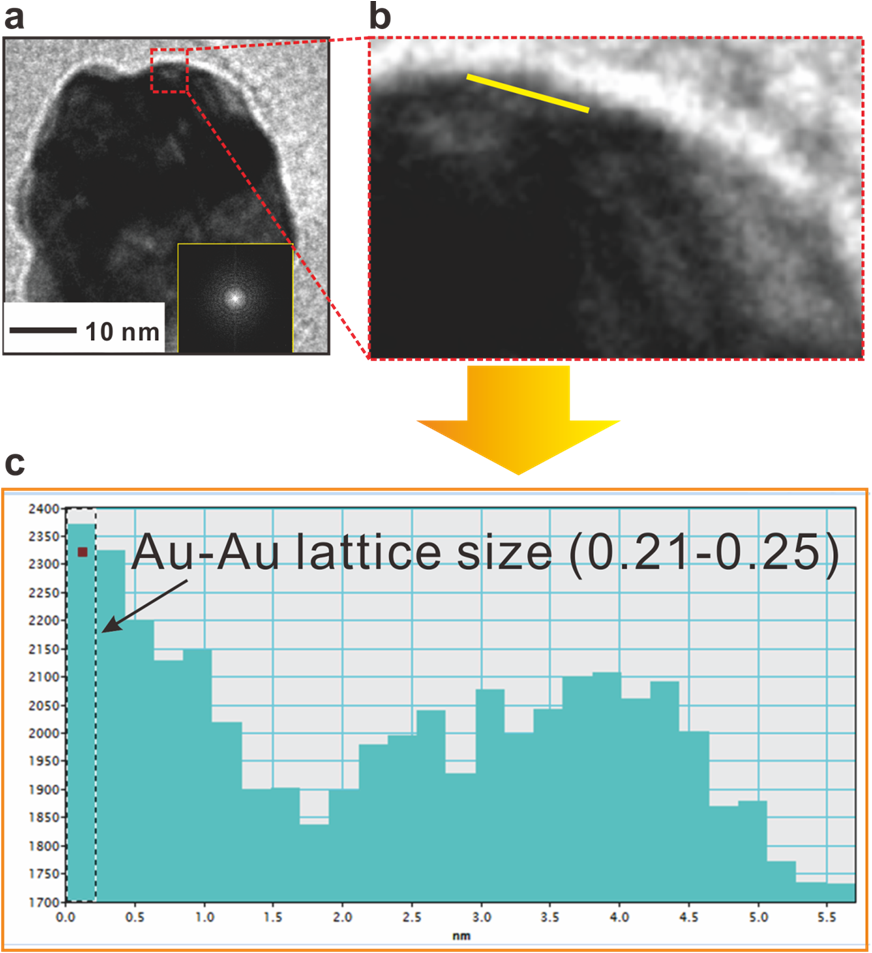
**

**Fig. S3.** HR-TEM analysis of (**a**) PEG-^124^I-Au@AuCBs (inset:FFTs [SAED patterns]). (**b**) Lattice plane of Au shellsshown in (**a**). (**c**) The distance of the lattice planes of Au shells in the marked region shown in (**b**).


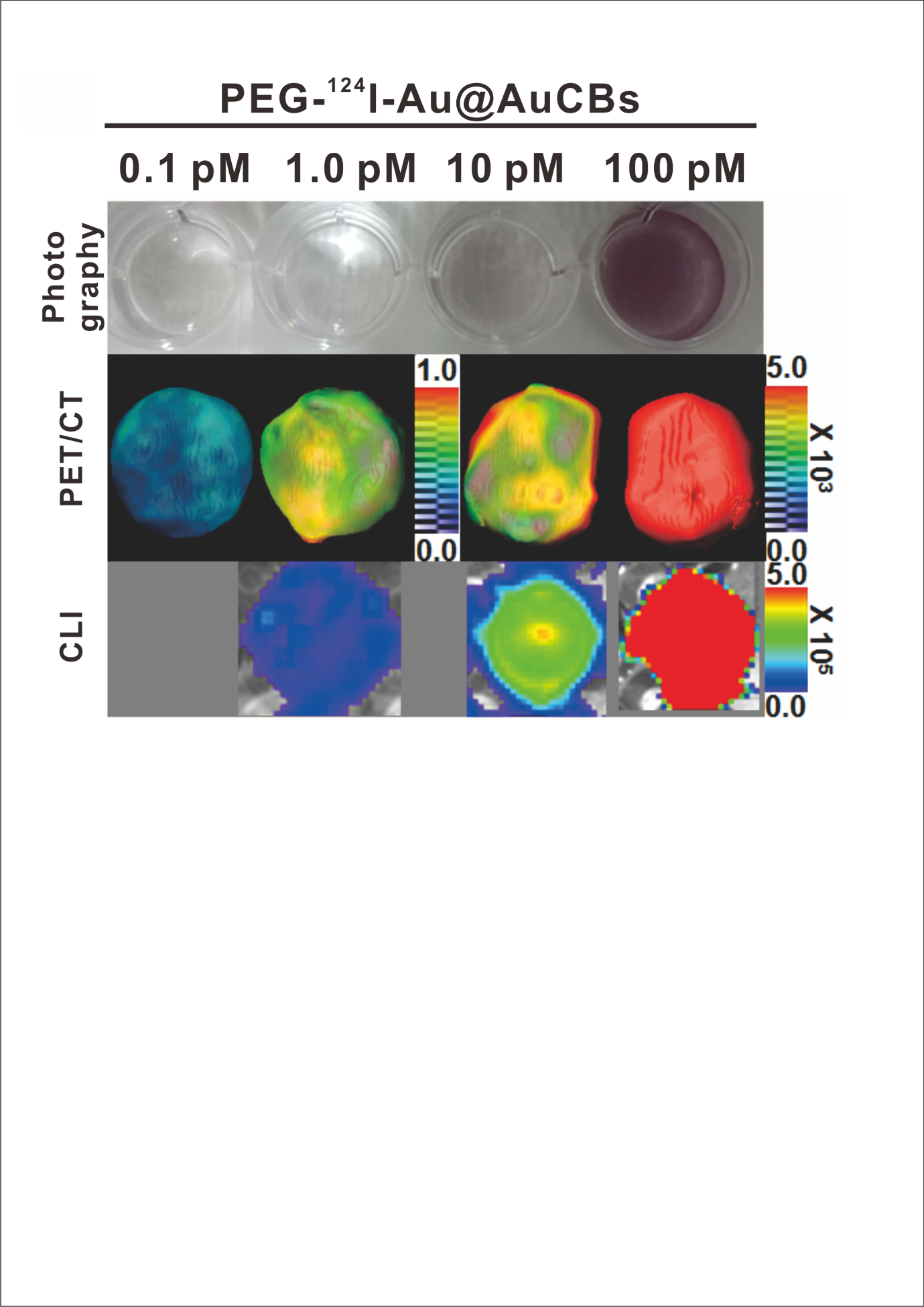


**Fig. S4. Sensitivity of PEG-^124^I-Au@AuCBs.** Photographs (upper), PET/CT images (middle), and CLI images (bottom) of the PEG-^124^I-Au@AuCB solution.


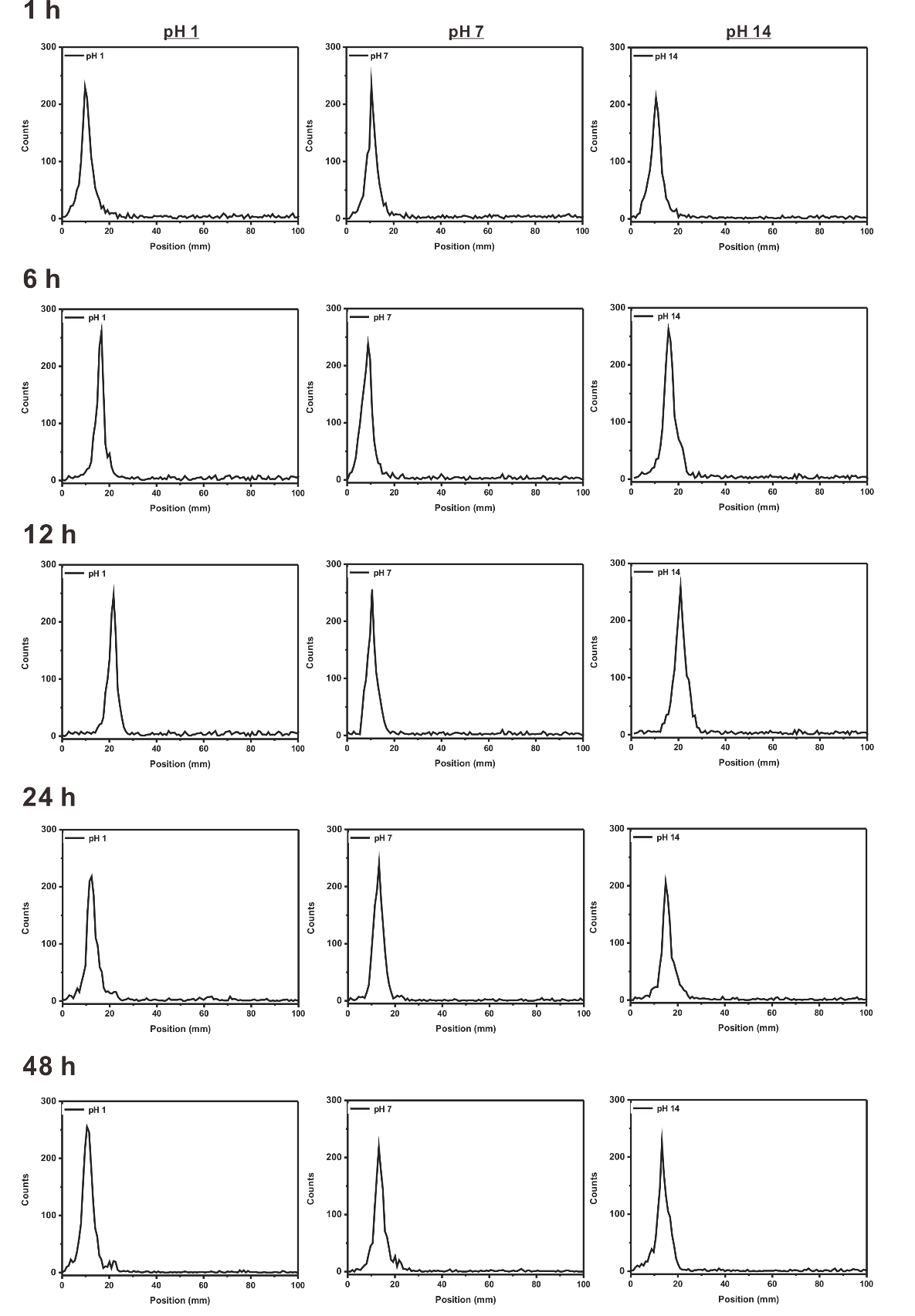


**Fig. S5.** Time-dependent stability of PEG-^124^I-Au@AuCBs in solutions of different pHs. The radioactivity released from particles was monitored using a TLC scanner.


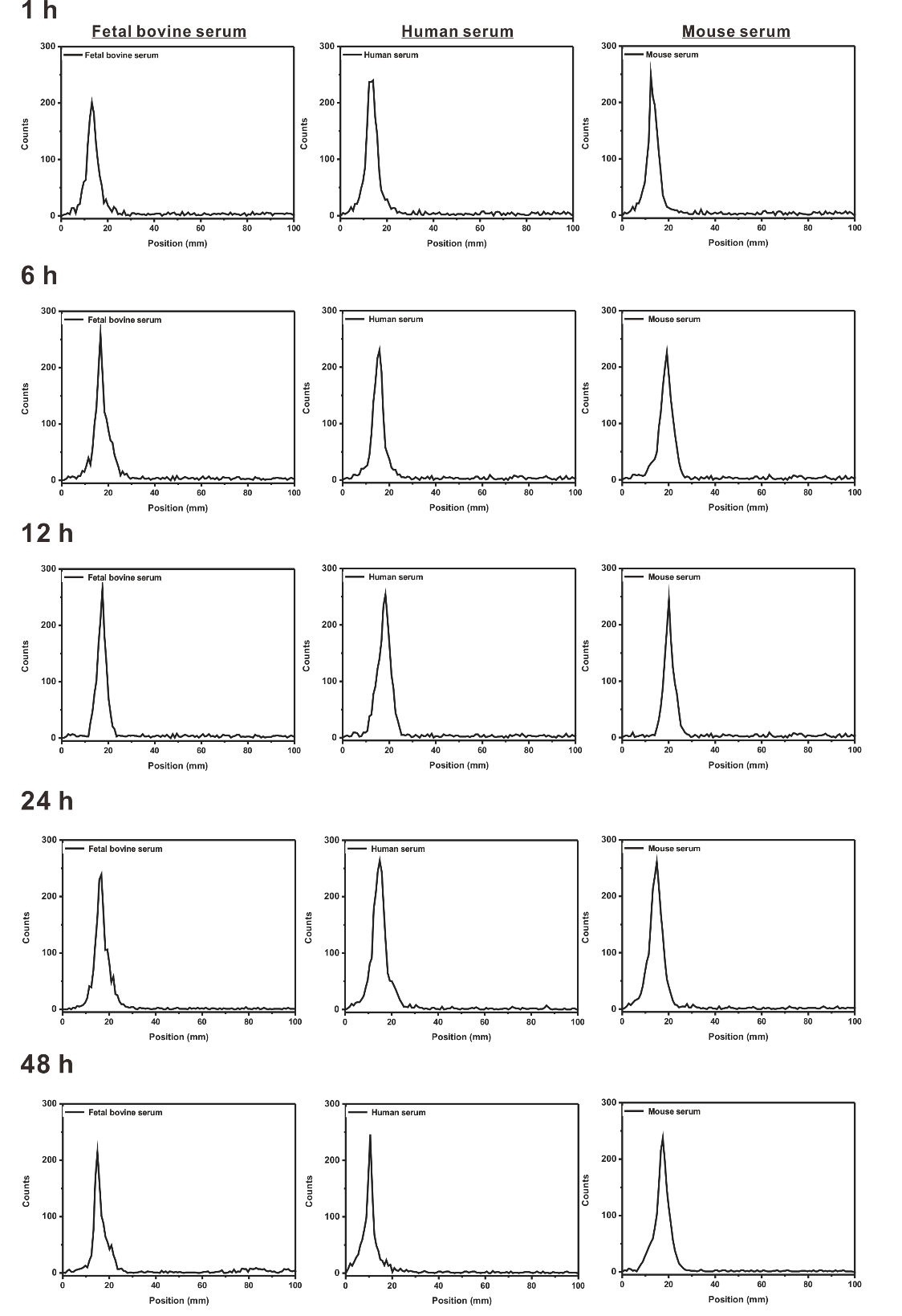


**Fig. S6.** Time-dependent stability of PEG-^124^I-Au@AuCBs in various sera, as monitored by TLC. The radioactivity released from particles was monitored using a TLC scanner.


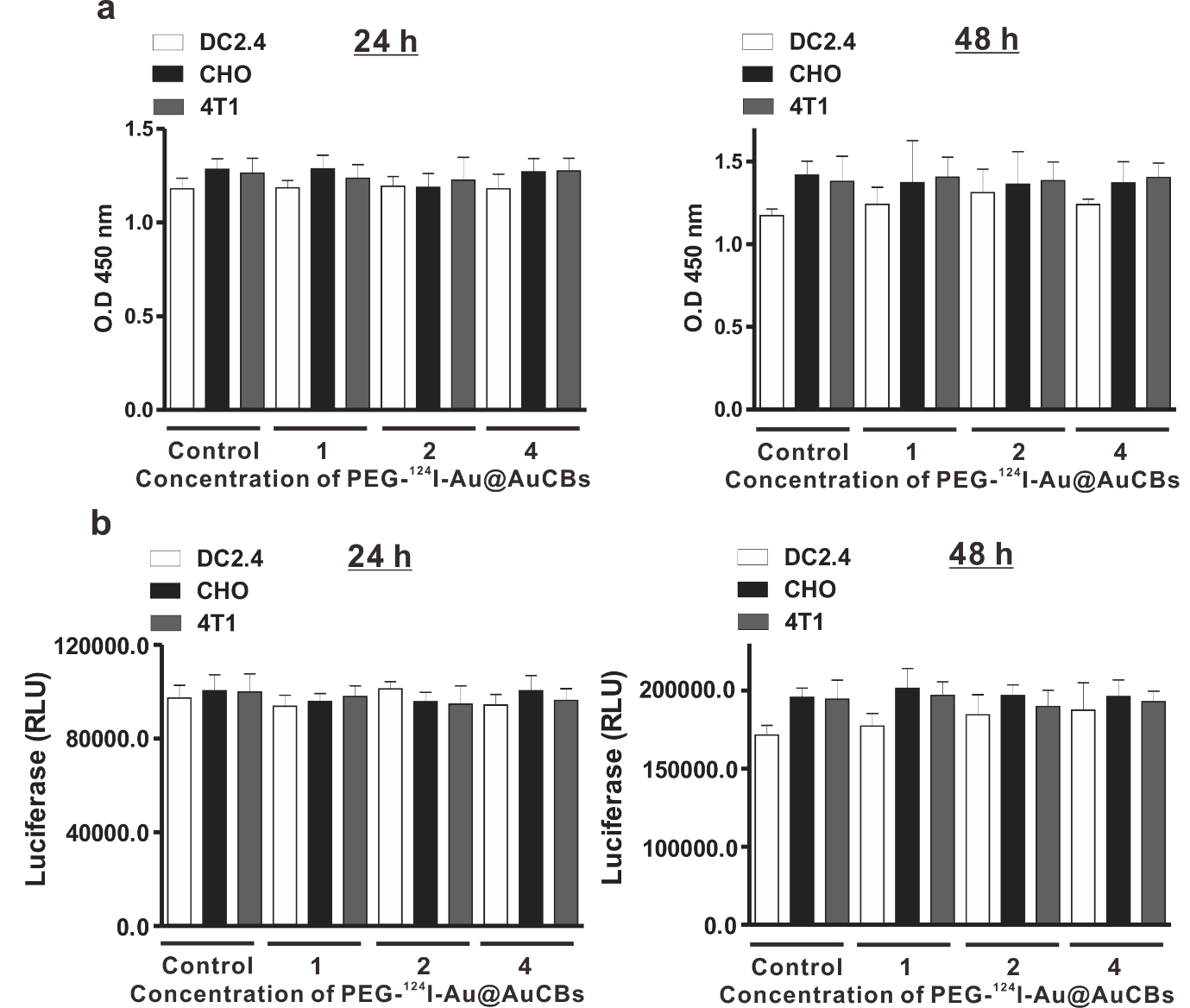


**Fig. S7.** Cell proliferation assay to determine the biocompatibility of ^124^I-Au@AuCBs in CHO, DC2.4, and 4T1 cells. Cells were plated in 96-well plates for 24 h, and PEG-^124^I-Au@AuCBs were then added for 48 h. Cell proliferation was determined with (**a**) Cell Counting Kit-8 (CCK-8) assays and (**b**) CellTiter-Glo® Luminescent Cell Viability Assay.


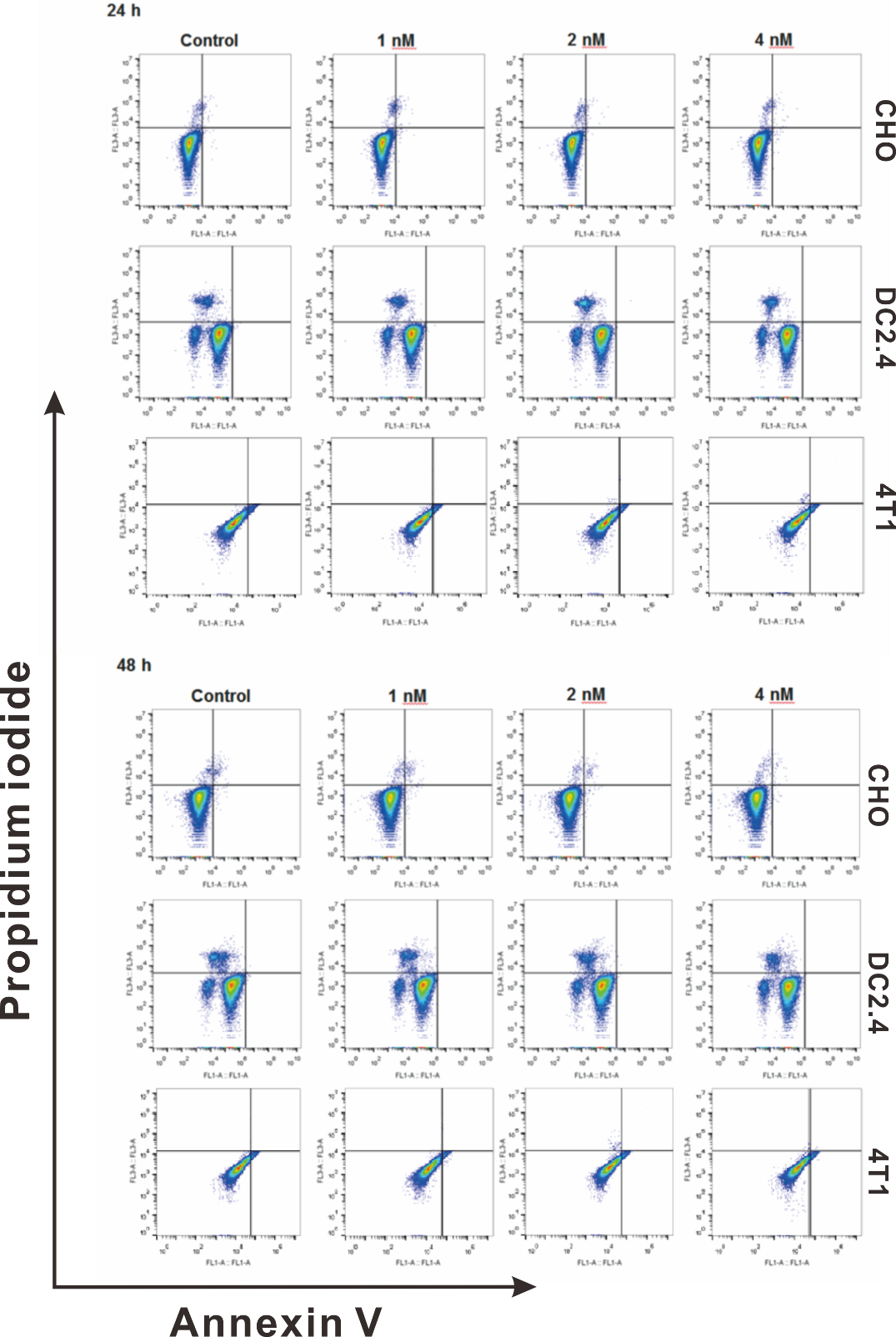


**Fig. S8.** Apoptosis analysis to determine the biocompatibility of PEG-^124^I-Au@AuCBs in CHO, DC2.4 and 4T1 cells. After incubating cells with PEG-^124^I-Au@AuCBs for 24 or 48 h, apoptosis levels were determined by annexin V and propidium iodide.


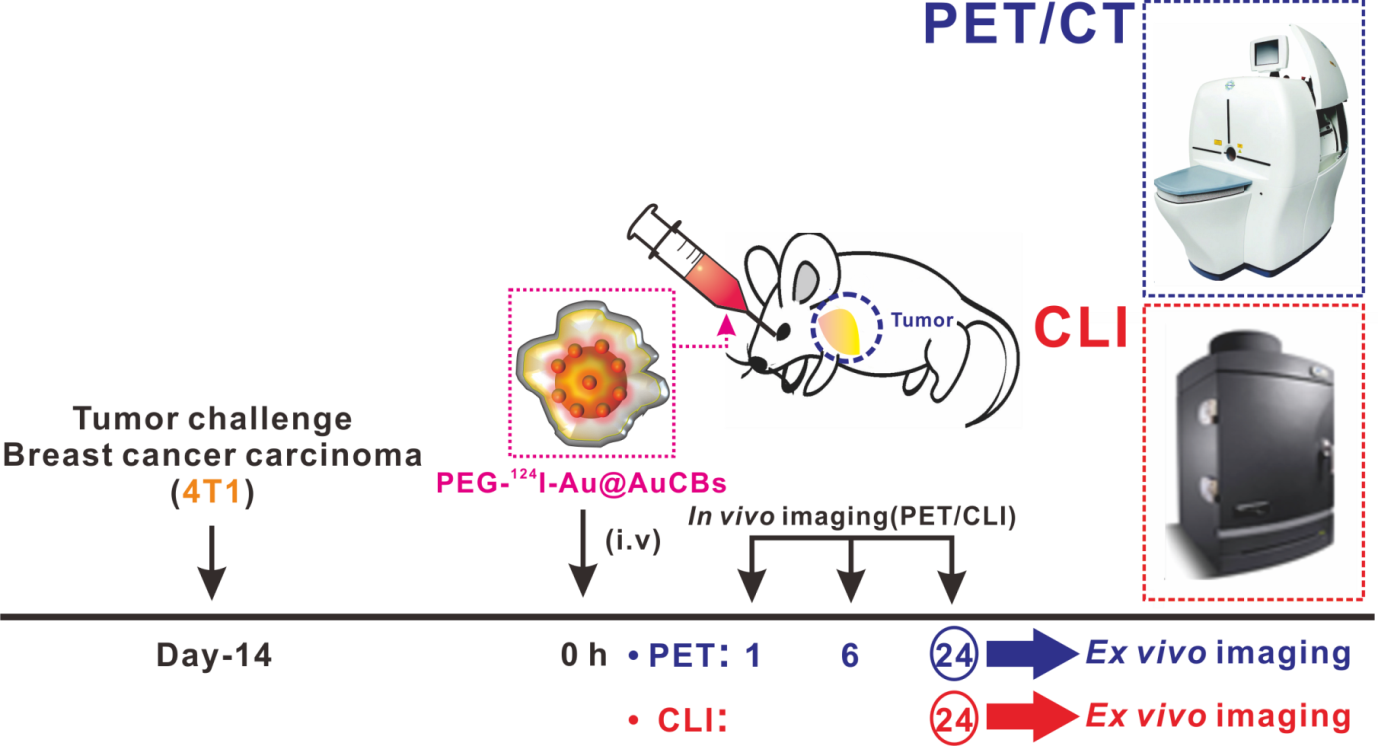


**Fig. S9.** Schematic procedures for *in vivo* tumor detection with PEG-^124^I-Au@AuCBs**.**Breast cancer-bearing mice received PEG-^124^I-Au@AuCBs via retro-orbital injection. Combined PET/CLI was conducted to determine the distribution of infused PEG-^124^I-Au@AuCBs at the indicated times. Ex-vivo PET and CLI was carried out at 24 h postinjection.


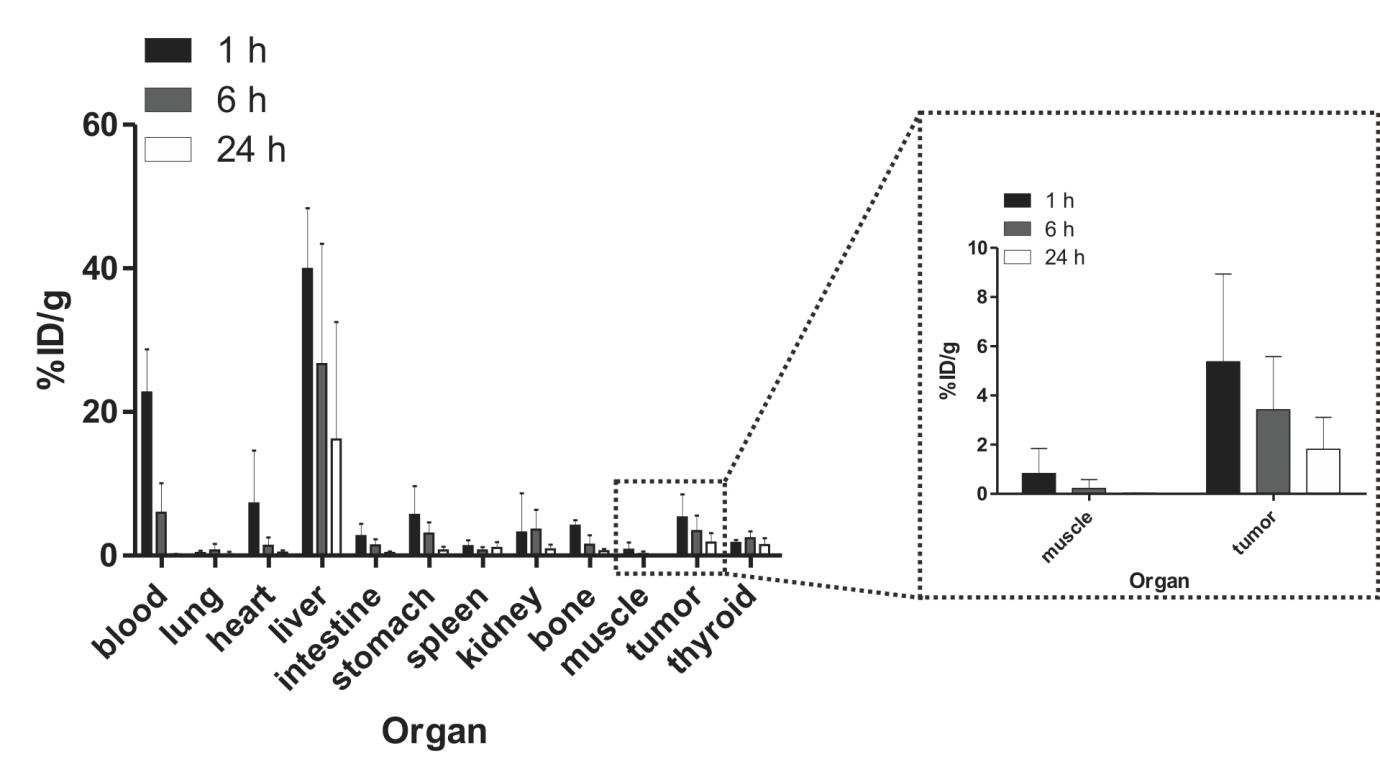


**Fig. S10.** Biodistribution of PEG-^124^I-Au@AuCBs in whole organs after retro-orbital injection. The black box shows a magnified image. %ID/g, percentage injected dose per gram.


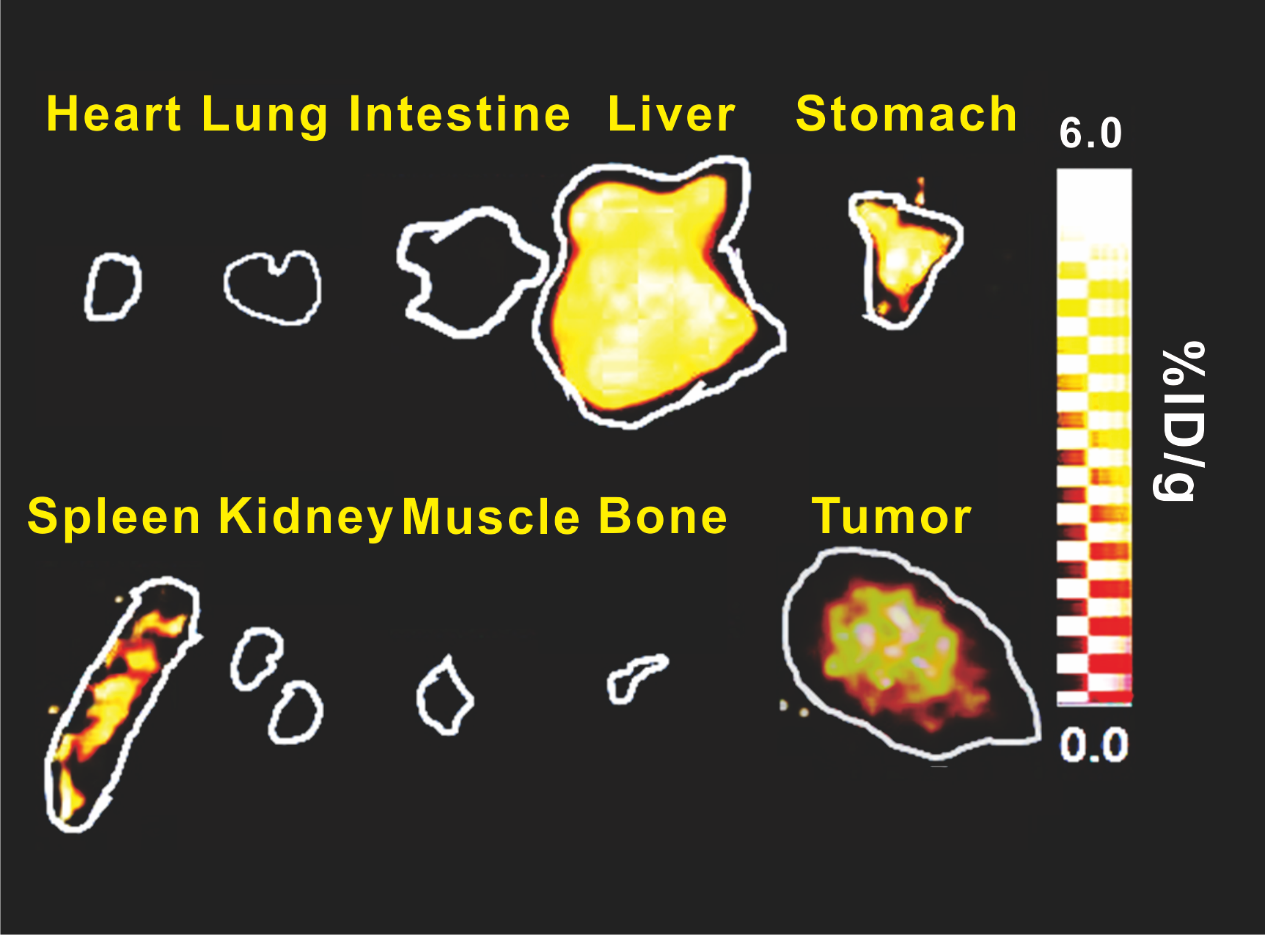


**Fig. S11.** *Ex-vivo* PET imaging of excised organs, including tumors.

**
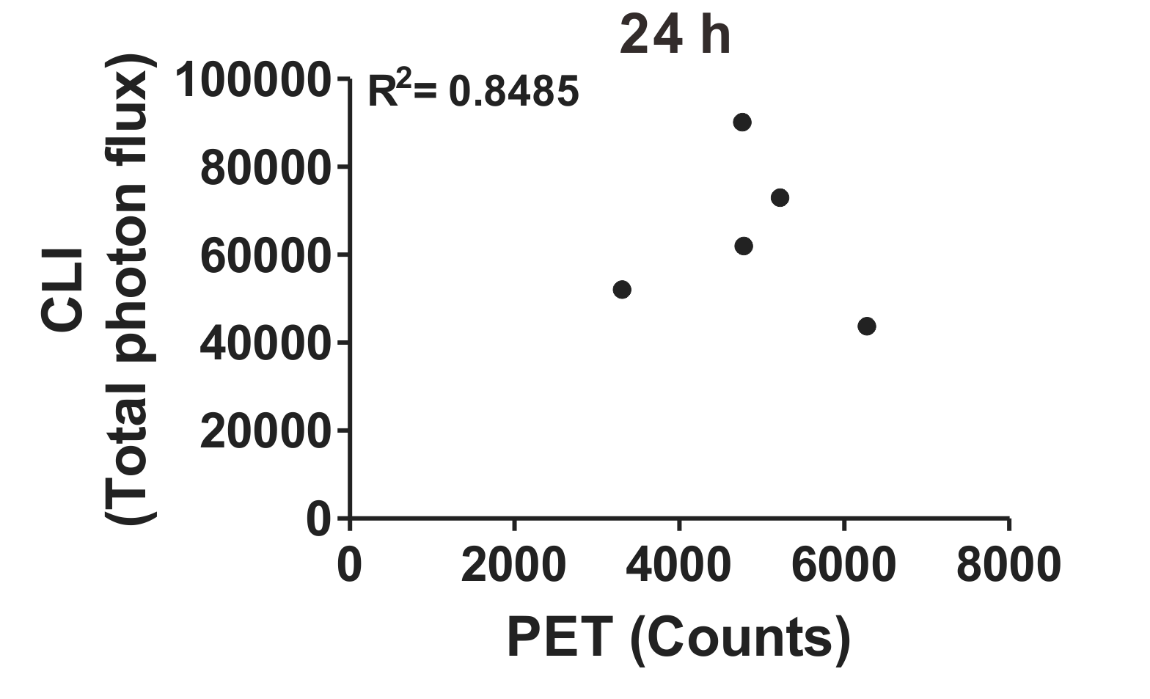
**

**Fig. S12.** Linearity between PET signals and CLI signals in breast cancer.


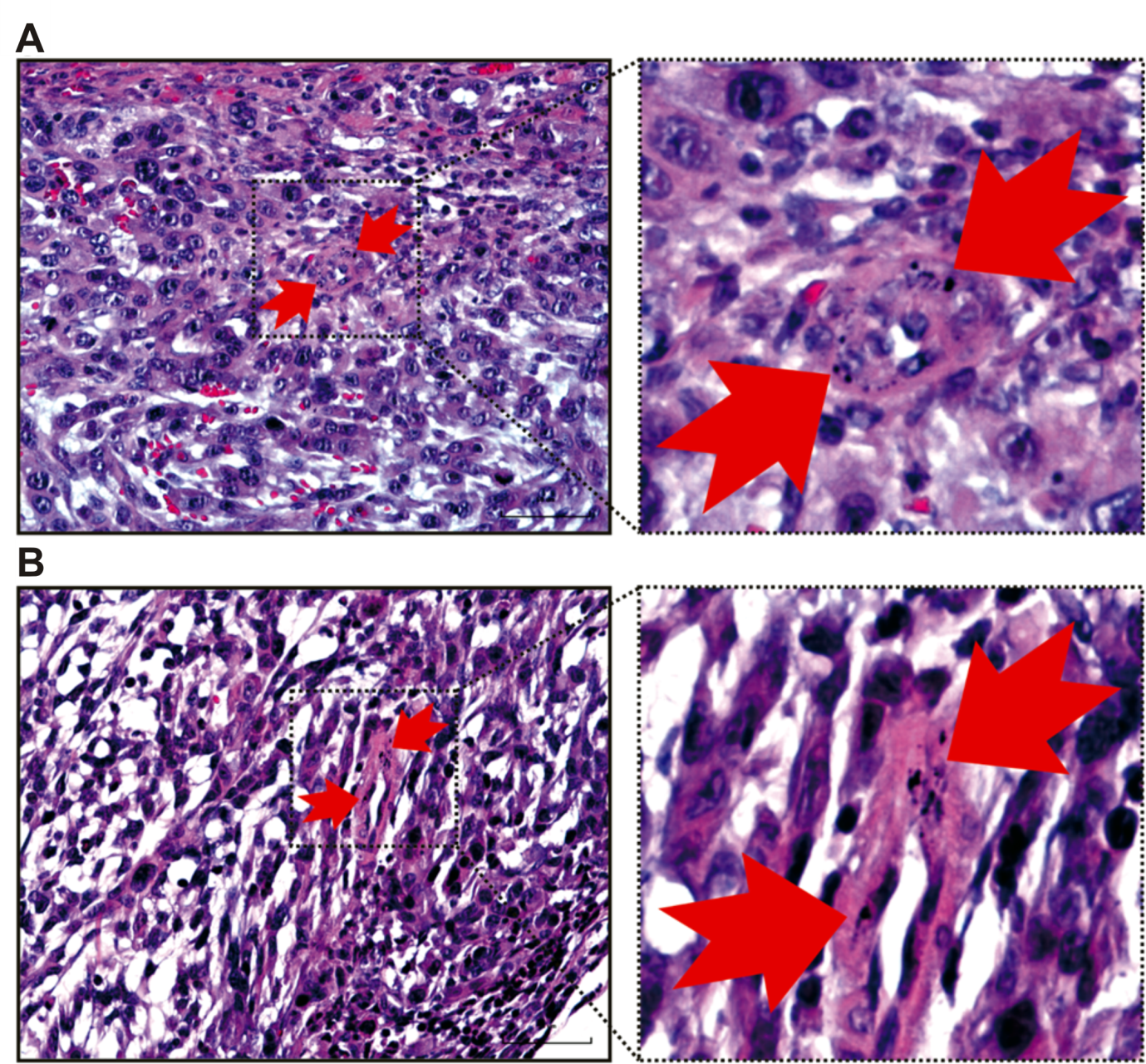


**Fig. S13. Histological analysis of breast tumors.** (**a**-**b**) Histological sections of tumor regions showing black nanocrushed ball-laden angiogenic vascular wall regions of the tumor. The red arrow indicates gold nanoparticles in the tumor.
